# User-Centered Design of Trauma Systems Solutions for Retriage of Patients With Injury: Mixed Methods Study

**DOI:** 10.2196/70846

**Published:** 2025-08-27

**Authors:** John Dwight Slocum, David Jelke, Qixuan Mai, Julie K Johnson, My L T Nguyen, Lixuan Cong, Adithya Chandrasekaran, Jane L Holl, Yuriy Moklyak, Justin Mis, Molly Gabaldo, James G Adams, William M Brigode, Mary Beth Voights, Rebecca Andersen, Tamuriat Gilbert, Nicole Siparsky, Arthur Proust, Anne M Stey, Andrew B L Berry

**Affiliations:** 1 Department of Surgery, Feinberg School of Medicine, Northwestern University, 420 E Superior St, Chicago, IL, 60611, United States, 1 (312) 503-8194; 2 Segal Design Institute, Northwestern University, Evanston, IL, United States; 3 Department of Surgery, University of North Carolina at Chapel Hill, , Chapel Hill, North Carolina, United States; 4 Center for Education in Health Sciences, Feinberg School of Medicine, Northwestern University, Chicago, IL, United States; 5 Center for Healthcare Delivery Science & Innovation, Department of Neurology, University of Chicago, Chicago, IL, United States; 6 Department of Surgery, Northwestern Medicine McHenry Hospital, Mchenry, IL, United States; 7 Department of Trauma and Burn, John H. Stroger, Jr. Hospital of Cook County, Chicago, United States; 8 Chicago Department of Public Health, Chicago, United States; 9 Department of Surgery, Carle Foundation Hospital, Urbana, United States; 10 Department of Emergency Medicine, Feinberg School of Medicine, Northwestern University, Chicago, IL, United States; 11 Department of Surgery, Rush University Medical Center, Chicago, United States; 12 Department of Emergency Medicine, Northwestern Medicine Delnor Hospital, Geneva, IL, United States; 13 Department of Medical Social Sciences, Feinberg School of Medicine, Northwestern University, Chicago, IL, United States

**Keywords:** user-centered design, re-triage, trauma system, bed capacity, feasibility

## Abstract

**Background:**

Retriage is the emergent interhospital transfer of severely injured patients from nontrauma and low-level trauma centers to high-level trauma centers. An estimated 17%‐34% of patients with traumatic injury are undertriaged to nontrauma or low-level trauma centers in the United States each year. These patients see 30% increased odds of mortality at 48 hours and nearly 4-fold increased odds of overall mortality. However, 30%‐50% of undertriaged patients are never retriaged to a high-level trauma center. Informatics-driven solutions facilitate time-sensitive exchange of patient information in other health care contexts. Few studies have explored how informatics-driven solutions can be tailored to address obstacles to timely, effective retriage.

**Objective:**

We applied user-centered design to develop a robust, acceptable, and feasible digital health solution to improve the retriage process.

**Methods:**

This was a mixed methods, observational, cross-sectional study. Potential frontline users of an intervention and hospital leadership were recruited to participate. Individuals in these roles included trauma medical directors, emergency department directors, trauma surgeons, emergency medicine physicians, emergency department nurse managers, emergency department nurses, trauma coordinators, emergency department bed managers, and health unit coordinators at nontrauma or low-level trauma centers and high-level trauma centers. We applied the 5-phase user-centered design approach, including phase 1: understanding the design needs, through site visit observations and focus groups; phase 2: ideation of potential solutions through a second round of virtual focus groups; phase 3: rank ordering solutions to identify the most robust, acceptable, and potentially feasible solutions; phase 4: prototyping by creating low-fidelity prototypes for the highest-ranked solutions; and phase 5: validation of the robustness of the prototypes through virtual focus groups. Validation approaches included asking frontline end users to assess the feasibility of each prototype and whether prototypes would address the identified retriage failures and barriers. In addition, leaders were asked to assess the feasibility of implementing the proposed solutions in their trauma center. All virtual sessions were recorded, transcribed, and inductively coded to generate themes of robustness, acceptability, and feasibility of the retriage solution. Thematic analysis was anchored on the desirability, viability, and feasibility design thinking methodology.

**Results:**

Nineteen sessions were conducted across all 5 phases with 49 participants from 12 trauma centers across Illinois. Participants included frontline users and leadership. The key design requirement was resource transparency between centers. The ideation phase produced 70 solutions. A systemwide bed tracker was ranked the highest by participants. Prototyping and validation resulted in a centralized, systemwide, bed tracker with hourly updated bed availability being the final solution to improve the retriage of patients with traumatic injury from non- or low-level trauma centers and high-level trauma centers.

**Conclusions:**

A 5-phase user-centered design approach resulted in a single solution consisting of a digital bed-tracker with frequently updated data on beds at high-level trauma centers to improve retriage.

## Introduction

Undertriage occurs when severely injured patients are taken from the field to nontrauma centers instead of high-level trauma centers [1]. Undertriaged patients experience 24% higher odds of mortality than patients taken directly to high-level trauma centers [2]. Retriage is the emergent transfer of undertriaged patients to high-level trauma centers [3]. Patients who are retriaged within 120 minutes of arrival at nontrauma or low-level trauma centers experience mortality similar to those brought directly to a high-level center [4]. However, 30%‐50% of undertriaged patients are never retriaged to a high-level trauma center [5]. Timely retriage of severely injured patients is limited by evaluations in the field and at nontrauma or low-level trauma centers before retriage and, then, by the ability to identify a bed available and receive acceptance of transfer to a high-level trauma center [3,6,7]. There are limited guidelines determining how retriage is carried out. Only 22 states have retriage guidelines, and clinical criteria for transfer vary widely [8]. In addition, states with trauma funding see higher retriage rates and lower in-hospital mortality than states without [9]. The retriage process itself can be summarized by two key phases when a severely injured patient is brought to a nontrauma or low-level trauma center: (1) identifying the patient’s care needs that trigger the transfer process and; (2) the transportation of the patient and their data to a high-level trauma center [6]. Informatics-driven solutions help facilitate complex processes in other health care contexts by streamlining the exchange of information between providers and flow of patient data across care systems. Specific solutions that have seen success include informatics-driven tools to facilitate patient triage during the COVID-19 epidemic and telemedicine to promote rural health care access [10,11]. There is a critical gap in literature exploring how informatics-driven solutions can be tailored to address obstacles to timely, effective retriage. The study objective was to use a user-centered design (UCD) approach that identifies design requirements for an acceptable and feasible informatics-driven solution to improve retriage between nontrauma or low-level trauma centers and high-level trauma centers in Illinois. Multidisciplinary end users and leaders involved in the process of retriage were engaged to take part in a UCD approach to generate and refine a solution for timely retriage. A wide range of end users (eg, frontline clinical and transfer) and leaders at both nontrauma or low-level trauma centers and high-level trauma centers participated in the 5-phase (understand, ideate, rank, prototype, and validate) UCD approach to create the solution. The study aimed to use these findings to articulate how informatics-based solutions could promote timely and effective retriage for the stakeholders and settings included in retriage processes across Illinois.

## Methods

### Study Design

**Figure 1.**
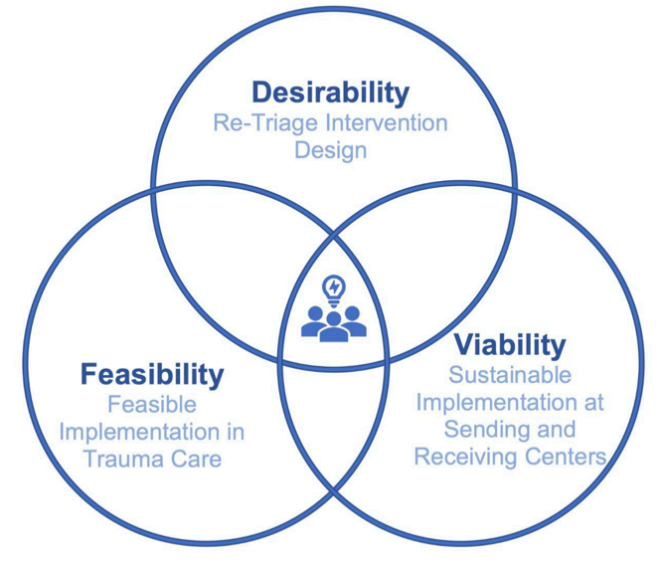
Desirability, viability, and feasibility design thinking methodological orientation of user-centered design approach for an observational cross-sectional mixed methods study of the retriage process with 10 nontrauma, low-level, and high-level trauma centers in Illinois 2022‐2023.

**Figure 2.**
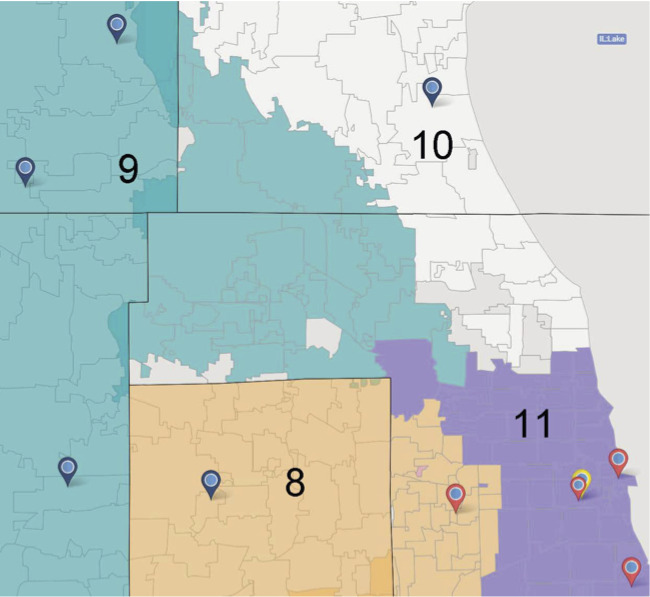
Study setting of nontrauma, low-level, and high-level trauma centers participating in user-centered design in Illinois 2022‐2023.

This was an observational cross-sectional mixed methods study. UCD was the main approach used. UCD is an iterative approach that involves all end users (and leaders) of a process or product in the entire design process of a solution [12,13]. It involves 5 phases (Figure 1) to understand the design needs, ideate potential solutions, rank order the solutions in relation to the identified design needs, prototype the highest ranked solutions, and then validate the solution prototypes by robustness, acceptability, and feasibility [14]. UCD is ideal for complex systems, with multidisciplinary end users and leaders from multiple organizations needing to interact to achieve a desired outcome [15]. UCD has increasingly been used in health care process and product design. The desirability, viability, and feasibility design thinking methodological orientation was used to analyze the data collected during the UCD process (Figure 2) [16]. The manuscript was written in adherence to the Consolidated Criteria for Reporting Qualitative Research (COREQ) checklist (Checklist 1).

### Setting

Clinicians and staff from 4 high-level (Level I), 5 low-level (Level II) centers, and 3 nontrauma centers in Illinois participated. Illinois is divided into 11 emergency medical services regions. Each region has at least one Level I trauma center serving as a “resource center” that has quarterly meetings with Trauma Medical Directors and Emergency Medical Services Directors in their region. Patients with severe injury are undertriaged from the field to nontrauma centers 17%‐34% of the time [17-19]. Illinois has 2 tiers of trauma center designation, based on adherence to structural requirements and standardized processes [20]. Illinois Level II trauma center designation requirements mirror those of the American College of Surgeons low-level centers (level III and IV) and Level I trauma center designation requirements mirror those of the American College of Surgeons high-level centers (level I and II) [20]. The terminology non-, low-, and high-level trauma center is used accordingly in this manuscript.

### Ethical Considerations

The Northwestern University institutional review board approved the study (STU00213470). Participants were consented before each session and informed of their ability to opt out at any time should they not want to continue with the interview. Data from each session was stored on a password-protected, encrypted server that was accessible only by members of the study team. Participants were not compensated for their participation in this initiative. No identification of individual participants has been made in the manuscript’s figures or supplementary materials.

### Research Team Reflexivity

Four authors JDS, DJ, MX, and AMS conducted the sessions. JDS is a project manager and PhD candidate, with a MPH, DJ, and MX are candidates for a MS in Design. AMS is a trauma surgeon scientist and MS in Health Policy. JDS and AMS had prior experience in conducting mixed methods studies. Two research team members had previously interacted with participants during a previous Failure Modes Effects and Criticality Analysis [21]. The research team’s personal goals, reasons for doing the research, and interests in the topic were delineated in the informed consent script at the start of each virtual session.

### Participant Recruitment

The study team initially recruited hospitals by contacting trauma coordinators and surgeons for each hospital listed in the Illinois Department of Public Health Continuous Quality Improvement Committee listserve. They also contacted surgeon champions and surgical clinical reviewers for each trauma center in the Illinois Surgical Quality Improvement Collaborative listserve. Each resource included urban, suburban, and rural high-level, low-level, and nontrauma centers across the state. Snowball and purposive sampling were used to complete the recruitment of participants. Frontline end users who provide direct care to severely injured patients were recruited. These included emergency medicine physicians, trauma surgeons, emergency department (ED) nurses, and ED para-professionals. The study team also recruited transfer end users who participate in key retriage tasks that include transfer coordinators, bed managers, and ED clerical staff. Participant leader end users included trauma medical directors, emergency medical services directors, trauma coordinators, ED nurse clinical coordinators, and quality improvement leaders. Personalized participation invitations were extended via email. An institutional review board approved informed consent was obtained from all participants, initially, and before each of the 5 phases.

### Data Collection

**Figure 3.**
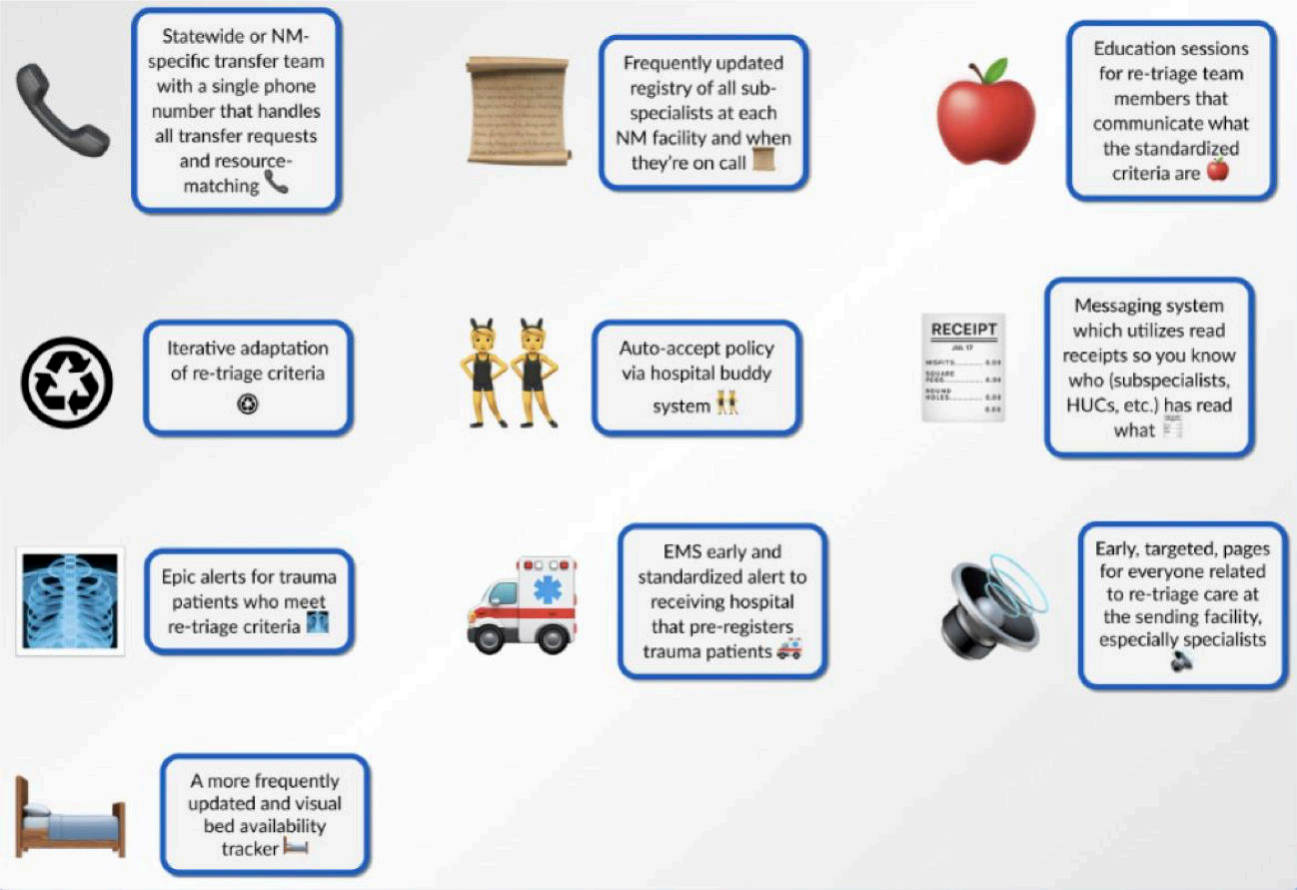
Candidate retriage solutions generated during user-centered design phase II—brainstorming solutions for the retriage process with 10 nontrauma, low-level, and high-level trauma centers in Illinois 2022‐2023.

Data collection occurred by video-conference sessions, during business hours. Data collection sessions were held between March 2022 and August 2023 (Figure 3).

#### Phase I: Understand

Phase I included focus groups and site visit observations to understand design requirements and constraints for retriage solutions. The phase I semistructured interview guide (Multimedia Appendix 1) mapped the retriage process and its failure points [6,21]. The interview guide was grounded in the desirability, viability, and feasibility design thinking methodological orientation [16]. Data collection occurred with each team from each center via 30-minute, virtual focus group sessions via videoconference. Teams at nontrauma and low-level trauma centers described the steps from arrival of a patient potentially eligible for retriage, either walk-in or by ambulance, through transfer of the patient to a receiving high-level center. High-level trauma teams defined steps from receiving the request for transfer of a retriaged patient from a nontrauma or low-level trauma center to arrival of the patient at the receiving high-level center for definitive care. The focus groups were audio recorded and transcribed verbatim. The intended scope of the design requirements was described as the concurrent and interrelated retriage requirements between the sending nontrauma or low-level trauma centers and receiving high-level trauma centers. Leadership defined the design constraints. 3-hour observations were conducted at each site by walking through the retriage process with trauma coordinators. Field notes were taken during the observations. Transcripts and field notes served as qualitative data for this phase. The central requirements for all steps from retriage start to retriage end were mapped. Results were used to determine design requirements from beginning to end of the retriage process. The requirements informed brainstorming in phase II.

#### Phase II: Ideation

Phase II consisted of a single 90-minute virtual focus group via videoconference in which study participants were encouraged to generate as many solutions as possible without imposing any constraints. The Phase II focus group interview guide was based on the IDEO Brainstorm Framework [22] using five how-might-we questions (Multimedia Appendix 2). Before the ideation session, participants were asked by email to list potential solutions broadly addressing the design needs from Phase I. During ideation, participants shared and categorized potential solutions using FigJam, a web-based design tool [23]. This process encouraged participants to generate a wide range of ideas (divergent ideation), after which the study team refined and synthesized overlapping ideas for solutions (convergent ideation). Participant responses to the interview guide during the focus group served as data collected through audio recording which was then transcribed verbatim. Transcripts and FigJam boards served as the qualitative data for Phase II. Results informed the solutions to rank in Phase III.

#### Phase III: Rank Ordering

The study team reviewed solutions from Phase II and eliminated those that were redundant or not adherent to Phase I design needs and constraints. A total of 10 solutions were presented for ranking along a 2-axis system anchored to the acceptability and feasibility framework of Campbell et al [24] for evaluating complex health interventions. Participants were assigned to one of 2 synchronous breakout rooms during the videoconference session. The rationale for the 2 breakout rooms was to create smaller groups (<8 participants) in which participants would feel comfortable speaking up. Each breakout group was facilitated by a study team member (JDS and DJ) and included both end users and leaders from different centers. Participants collectively ranked, on Figma boards, the solutions on the 2 axes of acceptability and feasibility [25]. The ranked solutions were then sorted by rank scores in descending order to identify the 3 highest-ranked solutions on acceptability and feasibility (right upper quadrant in 2-axis system) by participants in each session. Results informed the solution to prototype in Phase IV.

#### Phase IV: Prototype

Phase IV consisted of 3 iterative prototyping sessions conducted by the study team. Three solutions were selected for low-fidelity prototyping, based on phase III findings, and were represented as storyboards by DJ. Each storyboard depicted use of one prototype by clinical end users in multidisciplinary roles. AMS, JDS, JLH, and ABB reviewed the storyboards in three virtual sessions. Storyboards were modified to better represent realistic retriage process workflows, while adhering to the principles of process design [26]. The storyboards were finalized once no further feedback was offered by the study team (saturation). The three storyboards served as the qualitative data from phase IV. These results were then presented to all end users for validation in phase V.

#### Phase V: Validation

Participants engaged in a 30-minute semistructured focus group that was recorded and transcribed (Multimedia Appendix 3). Each storyboard prototype was presented to participants for feedback, aligning the prototypes with the retriage workflow at each center. The study team used separate semistructured interview guides for frontline end users versus leaders. Frontline end users were asked to assess the feasibility of each prototype and whether prototypes would likely address the identified retriage failures and barriers. Leaders were asked to assess the feasibility of implementing the proposed solutions in their trauma center. Sessions were conducted until saturation of feedback was achieved [27].

### Reflexive Thematic Analysis

The study team conducted reflexive thematic analysis beginning with data collected in Phase I and continuing through the completion of Phase V. Transcripts and field notes from each phase were inductively coded by 5 study team members (JDS, DJ, MN, LC, and AC) to develop an initial codebook. Two study team members coded each transcript to promote iterative reconciliation and assure confirmability and dependability throughout the coding process. When reconciliation could not be reached, a third study team member or senior author adjudicated. Codes were organized into a coding tree to identify themes in design requirements and participant preferences for retriage solutions. Potential themes were checked and rechecked throughout the coding process to promote reflexive understanding of emerging trends in the data among team members [28]. After coding and theme generation, the study team evaluated and sorted codes and quotes to generate retriage solutions [29] MAXQDA 2022 (VERBI Software, 2021) was used for data management and analysis [30]. Transcripts were not returned to participants. However, member checking was completed when findings from the previous phase were presented back to participants from high-level, low-level, and nontrauma centers for comment at the beginning of each subsequent phase [31]. Participants corrected the team’s interpretation of findings.

## Results

### Overview

A total of 49 frontline users and leaders participated in 19 sessions across the 5 phases (Table 1). A total of 18 participants attended more than 1 session.

**Table 1.** Participants' roles in the user-centered design approach of observational cross-sectional mixed methods study of the retriage process with 10 nontrauma, low-level, and high-level trauma centers in Illinois 2022‐2023.

User type and role	Participants, n
Leadership	
Trauma medical director	8
Emergency department director	2
Frontline users	
Trauma surgeon	15
Emergency medicine physician	3
ED^a^ nurse manager	4
ED bed manager	1
Trauma coordinator	13
ED nurse	2
Health Unit Coordinator	1

a ED: emergency department.

### Phase I: Understand

The understand focus group included 7 trauma coordinators, 6 surgeons, 4 emergency physicians, 3 nurses, and 1 bed manager, representing 10 trauma centers. In previous work, we mapped the retriage process between non-, low-, and high-level centers at a high-level trauma center and 2 low-level trauma centers. Low-level and nontrauma centers in Illinois cited obstacles to determining clinical indicators for initiating the retriage process and challenges identifying level I trauma centers with space to accept the patient as 2 key opportunities for improving retriage [6]. High-level trauma centers cited obstacles coordinating patient transportation and efficiently exchanging clinical and radiologic data between sending and receiving centers as intervention targets [21]. These findings provided the basis for the three main design requirements for this study: (1) match patient needs with trauma center resources through timely communication; (2) identify accepting high-level trauma center efficiently; and (3) streamline the transfer of patient data between sending and receiving centers.

### Phase II: Ideation

Focus groups with 10 participants from 3 low-level trauma centers included 2 trauma surgeons, 3 emergency physicians, 3 nurses, 2 trauma coordinators, and 1 health unit coordinator. Participants generated 70 solutions. Candidate solutions were then recategorized into 10 solutions by the study team, based on the 3 key design needs from Phase I (Table 2).

**Figure 4.**
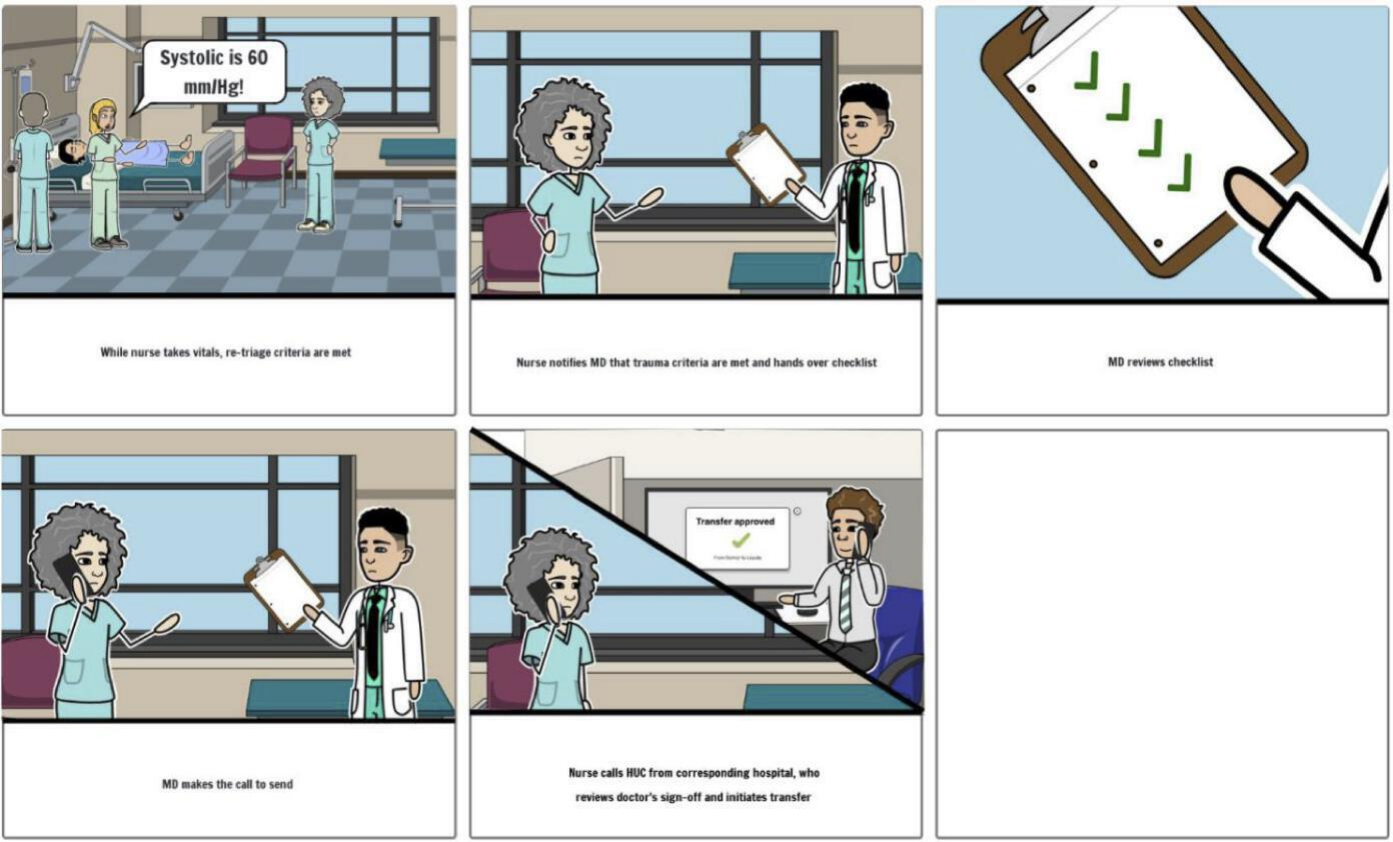
Checklist with standardized retriage criteria low-fidelity prototype from user-centered design phase IV—ranking solutions for the retriage process with 10 nontrauma, low-level, and high-level trauma centers in Illinois 2022‐2023.

Three potential solutions included a checklist, a bed tracker, and a transfer coordination center. The suggested checklist includes standardized retriage criteria for non-, low-, and high-level trauma centers. The checklist was proposed to help reach consensus about the need for retriage to help match patient needs with trauma center resources (Figure 4).

**Table 2.** Candidate retriage solutions generated during phase II—brainstorming solutions for retriage process with 10 nontrauma, low-level, and high-level trauma centers in Illinois 2022‐2023.

Intervention	Description
Transfer coordination center	Hospitals would call a single phone number to contact a team that handles all transfer requests and resource matching prior to and during retriage process.
On-call subspecialist registry	Nontrauma and low-level trauma centers would have a registry of subspecialists on call to help determine if a patient needs to be emergently transferred.
Retrige team education	Education sessions for retriage team members that communicate what standardized criteria are for initiating transfer of patients with severe injury.
Adaptation of standardized retriage criteria	Retriage criteria is updated based on changes to each hospital’s capacity to manage patients with severe injury.
Auto-accept policy	Nontrauma centers and low-level trauma centers establish partnerships ensuring that patients with severe injury needing retriage are automatically accepted at high-level trauma centers.
Retriage messaging system	Messaging system which uses read receipts so you know who has read which patient notes (eg, subspecialists and health unit coordinators).
Epic alerts for retriage candidates	Epic alerts specific to when a patient meets criteria to initiate retriage out of a nontrauma or low-level trauma center to a high-level trauma center.
Standardized alert for retriage patient arrivals	Standardized EMS^a^ alerts at high-level trauma centers thatpreregisters retriagepatientss when their arrival is imminent.
Retriage team pages	Early, targeted pages for everyone identified as a member of the retriage team at nontrauma or low-level facilities, including subspecialists who could be consulted.
Digital bed tracker	A frequently updated bed tracker accessible by hospitals statewide that shows current bed capacity at each nontrauma, low-level, and high-level center.

a EMS: emergency medical services.

*“There’s always gray area…Sometimes we feel that it’s an orthopedic injury that can be handled here at the [sending center]. However, the orthopedic [specialist] on call doesn’t feel comfortable with that type of injury and wants them transferred out.*

[Emergency Department Physician, Low-Level Trauma Center]

Bed availability at high-level centers changes rapidly and requires sending centers to call multiple high-level trauma centers before finding an available bed and receiving acceptance of the transfer. Therefore, the bed tracker would need to be updated frequently to provide non- and low-level trauma centers with accurate availability of beds at high-level trauma centers.

*[Bed availability] can change hour by hour… [statewide bed tracker] required updating bed status twice a day, and still, it was very inaccurate by the time we called for a bed…it would be even nicer if there was an electronic way of capturing so that it was more accurate and up to date in real time.*

[Trauma Coordinator, Low-Level Trauma Center]

A transfer coordination center could solve the need for a streamlined process of finding an accepting high-level center as well as assist in the sharing and transferring patient clinical data among non-, low-level, and high-level trauma centers. The transfer coordination center could reduce the multiple telephone calls to find an accepting high-level center while addressing conflicting opinions on patient needs requiring trauma center resources.

*… [city 1] is literally a two-minute drive from the [receiving center 1]. We will get transfers from them because [receiving center 1] either says ‘No, we’re not interested in it. This is a 70-year old who fell and has a questionable bleed, we’re too busy for that right now, send it to someone else.” So then [a sending center] will just move down the line.*

[Trauma Coordinator, High-Level Trauma Center]

#### Phase III: Rank Ordering

**Figure 5.**
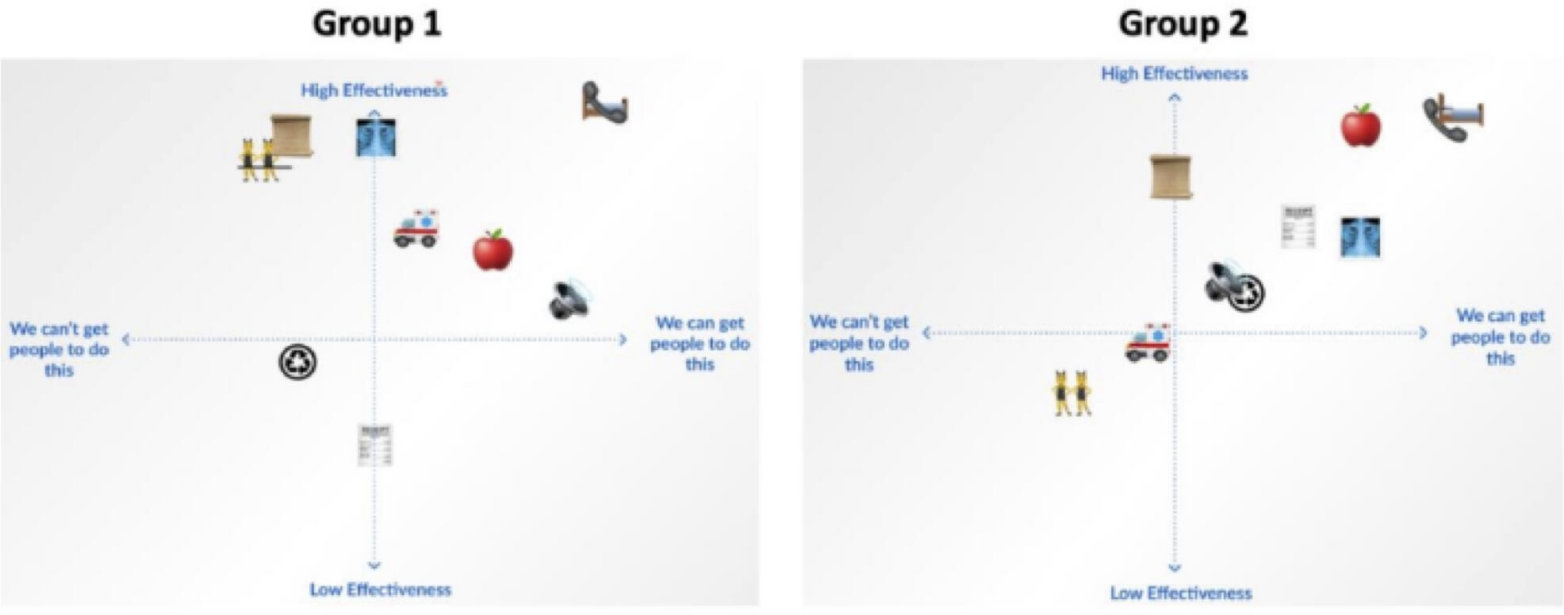
Retriage solutions on feasibility versus effectiveness axis from user-centered design phase III—ranking solutions for the retriage process with 10 nontrauma, low-level, and high-level trauma centers in Illinois 2022‐2023.

In total, 35 participants (clinicians, staff, trauma coordinators, and transfer coordinators) participated in the rank ordering activity (Figure 5).

#### Phase IV: Prototype

**Figure 6.**
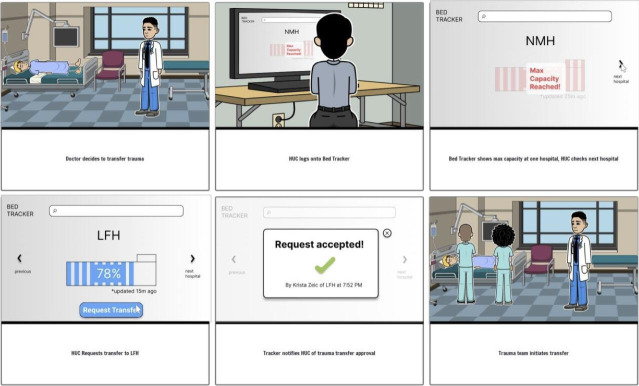
Bed-tracker low-fidelity prototype from user-centered design phase IV—ranking solutions for the retriage process with 10 nontrauma, low-level, and high-level trauma centers in Illinois 2022‐2023.

The research team developed low-fidelity prototypes for the 3 highest-ranked solutions. A storyboard format was used and iteratively developed, based on the three solutions in phase III by the study team (Figures4, 6  7).

**Figure 7.**
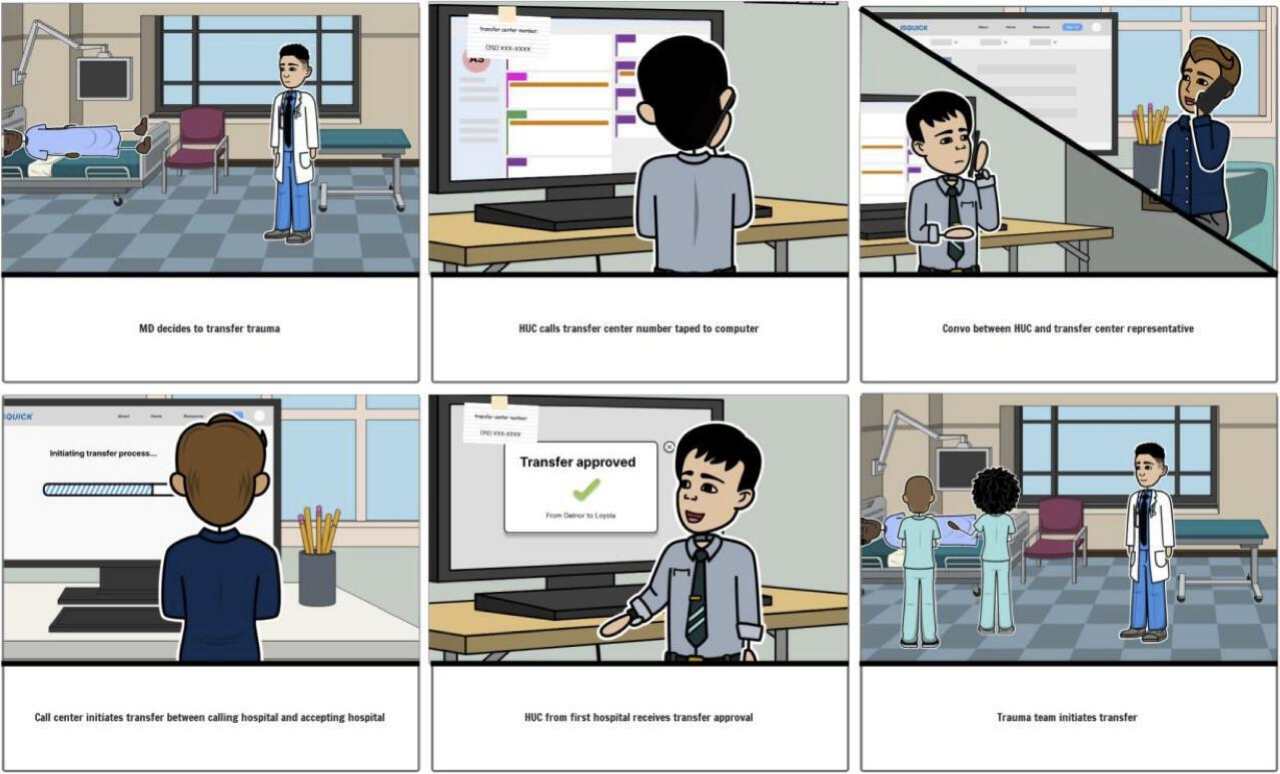
Transfer center low-fidelity prototype from user-centered design phase IV—ranking solutions for the retriage process with 10 nontrauma, low-level, and high-level trauma centers in Illinois 2022‐2023.

#### Phase V: Validation

The prototypes from Phase IV were presented for feedback to 15 frontline users from 2 level I trauma centers, 3 level II trauma centers, and 1 nontrauma center. Participants included 4 surgeons, 4 trauma coordinators, 3 emergency physicians, 2 ED nurses, and 2 bed managers. The reflexive thematic analysis of the group critique sessions produced a central theme, indicating that solutions exchanging time-sensitive resource information were most valuable.

The standardized retriage criteria checklist provided clarity about the appropriate trauma center level. However, most participants did not feel that retriage criteria would measurably improve the exchange of time-sensitive information, particularly at non- or low-level trauma centers that have less resources.

*If the patient is that severe and meets the checklist and gets transferred right as I’m walking in the door, then it’s sort of a waste of my time to drive in to see the patient because then the patient’s automatically just leaving…who’s doing the checklist and how is that going to change the role of the trauma surgeon in the community setting?...That’s what the state mandates…When it’s a Level 1, I have to drive in and examine the patient. That’s the whole point of trauma evaluation by a trauma surgeon in the trauma center. So I think a checklist is great, but then it’s not supposed to be the end all be all.*

[Trauma Surgeon, Low-Level Trauma Center]

Participants assessed that the bed tracker could promote shared understanding of real-time bed availability.

*…it’s up to the onus of the [high-level center] Emergency Room to be like, okay, even if we take this transfer and say they need an Intensive Care Unit, do I have an Intensive Care Unit to send them to? Or if it’s a gunshot wound that’s got a chest tube and is getting blood, sounds like they’re going to need an Operating Room? It’s a lot of working pieces, determining can we take a patient or not.*

[Bed Manager, High-Level Trauma Center]

A hospital resource management data platform owned by JUVARE and contracted by the Illinois Department of Public Health and the City of Chicago Department of Public Health.A bed tracker, based in the Chicago Department of Health, already existed, although underused and, was felt to be the most feasible solution, requiring only more frequent updating.

A “regional” Transfer Coordination Center was perceived as a solution to address the perceived lack of accountability for the exchange of retriage patient information. Without a transfer coordination center, the attending physicians at high-level centers have to assess the center's clinical capacity to coordinate urgently, as described below:

*[Non-clinicians] might not necessarily know about all the [subspecialty perspectives on a retriage candidate], whereas a clinician, when they get on call, they can make it part of their routine of like, hey, let me just check in with my neurosurgery, ortho and my hand guys to see if we’re open for this. And if they say, yeah, and we have beds then yes, we’re willing to accept it.*

[Emergency Department Physician, High-Level Trauma Center]

However, participants were unsure about the funding, management, and oversight of a “regional” Transfer Coordination Center. Although this prototype had the most participant excitement, enthusiasm was tempered by feasibility concerns.

### Thematic Analysis

The study team conducted reflexive thematic analysis of transcripts across all 5 phases of the study. The analysis yielded 16 themes that provided the basis for the design requirements and identification of potential solutions to retriage obstacles (Multimedia Appendix 4).

## Discussion

The objective of this study was to identify robust, acceptable, and feasible solutions to improve retriage between nontrauma or low-level trauma centers and high-level trauma centers in Illinois [6,21].

### Principal Results

This study found that a centralized, frequently updated bed tracker was the most effective and feasible informatics-based retriage solution. Within Illinois, the feasibility and potential effectiveness of a bed tracker were aided by the existence of hospital resource management data platform accessible to all hospitals across the state. However, the dashboard is underused. A future direction for further development of this solution requires encouraging use of the hospitals' resource management data platform.

Promoting increased uptake of the bed tracker among nontrauma, low-level, and high-level centers can enable Illinois’s trauma system to more effectively use real-time data to streamline the retriage process. In other states, bed trackers serve a crucial function of Medical Operations Coordination Centers, a model that helps trauma centers coordinate information exchanged between hospitals during transfer of severely injured patients [32,33]. States like Arkansas and Oregon use Medical Operations Coordination Centers available to all hospitals that provide frequently updated bed availability data, which has contributed to improved rates of timely, effective retriage in each state [33,34]. Our UCD process produced similar design requirements, adding valuable confirmatory evidence about the requirements for managing information associated with identifying the need for retriaging the resources available for meeting those needs.

Illinois’s trauma system is unique from most other states because it only has Level I, Level II, and nontrauma centers, rather than Levels I-IV, meaning that Illinois trauma centers at each level are uniquely heterogeneous in their capacity to care for severely injured patients [20]. However, our Failure Modes and Effects analysis of low-level or nontrauma centers identified that finding a receiving hospital with available beds was one of the most significant challenges, and there was demand for more effective tools to complete this step [6]. This clarifies the transferability of our study’s findings: applying informatics tools such as bed availability for trauma retriage in different locales (eg, US states) requires considering the heterogeneity in how different locales segment different levels of trauma care needs.

### Comparison With Prior Work

Informatics solutions have become common tools for improving care processes and access in health care contexts that require time-sensitive information exchange. While the retriage process depends on efficient coordination of resources and data exchange between hospitals, there is limited evidence exploring design requirements for informatics solutions in this context. This study is notable for its use of UCD to generate design requirements for an informatics solution that are responsive to retriage obstacles in a complex and heterogeneous trauma system. Informatics solutions that address complex processes in healthcare include approaches to equipment allocation in critical care medicine [35], patient triage during COVID-19 [36], and telehealth to improve rural trauma care access [37]. In addition, the UCD approach has been used to develop solutions for complex health care processes involving large, multidisciplinary teams [38], including hospital rounding [39] and aligning cancer treatment goals and progress between care teams [40]. Our study provides further support for the UCD approach in trauma system design while filling a gap in understanding around elements an informatics solution must have to improve retriage. It also suggests that broader adoption of UCD may enable systems to identify robust, acceptable, and feasible solutions to address complex challenges.

### Limitations

There were several limitations to this study. First, this analysis was prone to sampling bias. Most participants represented centers in a single urban geographic area, hindering the generalizability. However, the study team mitigated this bias by purposefully sampling stakeholders at each trauma center included in two statewide committees, which both include centers from more sparsely populated geographic areas. Second, the UCD approach is prone to detection bias because it relies on participant perceptions of processes or events that include subjective views. We sought to mitigate this bias by engaging a broad range of frontline users and leadership. Third, this study relied on participants to identify barriers in their hospitals' retriage process. This could have introduced recall bias. We mitigated this bias by holding multiple meetings with participants aimed at establishing trust necessary to properly identify design requirements for retriage solutions. Fourth, most interviews and focus groups were conducted virtually due to geographic barriers and COVID-19 restrictions at participating centers. However, the study team was able to leverage the virtual format by using a web-based design interface tool Figma that allowed participants to collectively complete exercises virtually.

### Conclusions

This study leveraged UCD to create a centralized, frequently updated bed tracker as the most likely robust, acceptable, and feasible retriage solution. Future directions will focus on refinement of the solution design and implementation.

## Supplementary material

10.2196/70846 Multimedia Appendix 1 Semistructured interview guide.

10.2196/70846 Multimedia Appendix 2 Brainstorm discussion guide.

10.2196/70846 Multimedia Appendix 3 Critique discussion guide.

10.2196/70846 Multimedia Appendix 4 Thematic analysis.

10.2196/70846 Checklist 1 COREQ checklist.

## ABBREVIATIONS

COREQ: Consolidated Criteria for Reporting Qualitative Research
ED: emergency department
UCD: user-centered design
