# Supplementary material for: User-Centered Design of Trauma Systems Solutions for Retriage of Patients With Injury: Mixed Methods Study
Source: J Med Internet Res. 2025 Aug 27;27:e70846. doi: 10.2196/70846 (PMC12381891; doi:10.2196/70846)
Supplement: Multimedia Appendix 1 [file jmir-v27-e70846-s001.docx]

**MULTIMEDIA APPENDIX 2: Semi-Structured Interview Guide**

Hello. My name is_______ and I am a ______, working with trauma surgeon Dr. Anne Stey on a project aimed at improving the trauma transfer and decrease the time it takes to initiate the process. High time to re-triage and undertriage rates are associated with higher mortality rates.

We have conducted research that examines the re-triage process at Illinois low-level, high-level, and non-trauma centers, and we are looking to pivot into a design phase. As a stakeholder with a critical perspective into the re-triage process at your hospital, we would love the opportunity to hear your insight.

Our goal is to gain additional insight into the re-triage process at your hospital, what obstacles you face when transferring a severely injured patient, and what determines the decision to initiate their transfer.

This session is intended to be on Zoom and last approximately one hour.

As described in the informed consent, we will audio-record and transcribe the interview, and remove all identifying information to protect your privacy and confidentiality. There are no right or wrong answers. We are interested in your perspectives on the steps in the process at your hospital, as well as what obstacles or ‘pain points’ exist. You may pause or stop taking part in interview at any time. Do you have any questions for me before we begin?

- First, tell us about your role(s) and day-to-day responsibilities at your hospital.
  - Probe: How do those responsibilities change from day-to-day?
- What is your involvement with the trauma transfer process for severely injured patients at your hospital?
  - Probe: How often do you come into contact with a severely injured patient that requires emergent transfer?
- What are some of the biggest obstacles you have observed in transferring patients between non-trauma/low-level trauma centers to high-level trauma centers?
  - Probe: Tell us more about those obstacles. What underlying factors contribute to those challenges emerging in the first place?
- What is the role of specialty consultants in determining whether to initiate the transfer process?
- What factors contribute to a smooth transfer of a severely injured patient from a low-level/non-trauma center to a high-level trauma center?
- If you could improve one thing about the current trauma transfer process, what would it be?
- Tell us about solutions, or workarounds, to some of the obstacles to timely, effective trauma transfers that you identified. How have those challenges been avoided or alleviated when transferring a severely injured patient?
- Who else should we speak with? What other roles do you feel have a perspective into the
