# Supplementary material for: User-Centered Design of Trauma Systems Solutions for Retriage of Patients With Injury: Mixed Methods Study
Source: J Med Internet Res. 2025 Aug 27;27:e70846. doi: 10.2196/70846 (PMC12381891; doi:10.2196/70846)
Supplement: Multimedia Appendix 2 [file jmir-v27-e70846-s002.docx]

**MULTIMEDIA APPENDIX C: BRAINSTORM DISCUSSION GUIDE**

Hello. My name is_______ and I am a ______, working with Dr. Stey on a project aimed at identifying opportunities to develop an intervention that promotes timely, effective re-triage of severely injured patients. High time to re-triage and undertriage rates are associated with higher mortality rates. We need your help and perspective to generate as many ideas as possible that stand a chance of solving the problem.

The purpose of this session is to have a more diverse set of stakeholders, specifically the “boots on the ground” individuals, to come up with as many ideas as possible based on a set of ‘How Might We’ (HMW) questions from the core brainstorm. Think of it just like our smaller core brainstorm, but with a larger group of people.

Our goal is to generate a wide range of potential solutions, as well as clarity as to which kinds of ideas are likely to be adopted and accepted by NM trauma workers.

Before getting started, we want to set some ground rules for this session. Today’s discussion will be framed by the 7 rules of brainstorming:

- - - - Defer judgment
      - Encourage wacky ideas
      - Build on the ideas of others
      - One conversation at a time
      - Stay on topic
      - Be visual
      - Go for quantity

This session is intended to be on Zoom and last one hour. This session will be held on Zoom, and we will use a virtual brainstorming platform called FigJam.

As described in the informed consent, we will audio-record and transcribe the interview, and remove all identifying information to protect your privacy and confidentiality. There are no right or wrong answers. We are interested in your perspectives on the factors that helped or hindered communication about the patient. You may pause or stop taking part in interview at any time. Do you have any questions for me before we begin?

Brainstorm: Set a timer for 10m for each ‘How Might We’ question and begin brainstorming.

- - - - HMW decrease the time it takes for consultants to call back the trauma team?
      - HMW overcome the issue of trauma subspecialty consultants not being available in a timely manner?
      - HMW optimize a process to determine receiving hospital bed availability?
      - HMW increase the likelihood that a receiving hospital accepts the patient?
      - HMW decrease the time it takes for teleradiologists to get back with image results?

Conclusion

- - - How did that exercise feel?
    - How do the interventions look? Which ones stand out as particularly feasible/acceptable?
