# Supplementary material for: User-Centered Design of Trauma Systems Solutions for Retriage of Patients With Injury: Mixed Methods Study
Source: J Med Internet Res. 2025 Aug 27;27:e70846. doi: 10.2196/70846 (PMC12381891; doi:10.2196/70846)

**MULTIMEDIA APPENDIX 4: CRITIQUE DISCUSSION GUIDE**

Hello. My name is_______ and I am a ______, working with Dr. Stey on a project aimed at identifying opportunities to develop an intervention that promotes timely, effective re-triage of severely injured patients. Trauma re-triage is often called trauma transfer by frontline workers. What makes trauma re-triage different is that it refers specifically to the emergency interfacility transfer of trauma patients at risk of mortality or disability from non-trauma or low-level trauma centers to high-level trauma centers.

The purpose of today’s session to update our understanding of the trauma transfer process and gain useful feedback from users to make more desirable and feasible prototypes. We’ve spoken with frontline trauma workers from several Illinois hospitals about how to properly provide timely effective re-triage. Then we created three low-fidelity prototypes that convey some potential solutions to problems we’ve heard. In this talk, we’d love to hear about your experience with trauma re-triage and get your feedback on these solutions.

Please give us your honest feedback. As described in the informed consent, we will audio-record and transcribe the interview, and remove all identifying information to protect your privacy and confidentiality. There are no right or wrong answers. We are interested in your perspectives on the factors that helped or hindered communication about the patient. You may pause or stop taking part in interview at any time. Do you have any questions for me before we begin?

**Questions for Nurse/Health Unit Coordinator:**

- How and where do you currently record results from the primary/secondary survey (eg. radiology, blood pressure, etc.?) Do you use Epic?
- How do you communicate survey results to the MD?
- Imagine that you have a list of criteria that, if met, automatically indicated that you needed to transfer a trauma patient to a higher level of care (eg. blood pressure, etc.). How would you want to record that information, and how would you want to communicate it to the trauma surgeon?
- [Present digital and physical checklist storyboards]
- **Digital Checklist Storyboard:**
- How would this concept work with your current process? What do you like about it, and what do you wish would be different?
- What do you think of this re-triage criteria checklist? What do you think generally about the idea of having a trauma criteria checklist?
- Once the trauma team decides it needs to transfer a trauma patient, how do you select a receiving hospital?
- **Bed Tracker Storyboard:**
- How would this concept work with your current process? What do you like about it, and what do you wish would be different?

**Questions for Trauma Surgeon/Emergency Department Physician:**

- What is your relationship to trauma re-triage? (role, experience)
- Who do you consider to be a part of your trauma re-triage team, and what are their roles?
- What’s your system for how you determine whether or not to transfer a trauma patient to a high-level trauma center?
- Imagine that you have a list of criteria that, if met, automatically indicated that you needed to transfer a trauma patient to a higher level of care (eg. blood pressure, etc.). What information would you need to see? How would you want to receive that information from a trauma nurse/support staff?

**Physical Checklist Storyboard**

- How would this concept work with your current process? What do you like about it, and what do you wish were different?
- What do you think about the specific trauma re-triage criteria listed?
- The next two solutions don’t involve the MD directly, but impact the overall process of re-triage, so we’re still interested in your thoughts.

**Digital Checklist Storyboard**

- How would this concept work with your current process? What do you like about it, and what do you wish were different?
- What do you think about the advantages and disadvantages of a physical vs. digital checklist?
- What’s your system for how your team currently identifies a receiving hospital for re-triage?

**Bed Tracker Storyboard**

- How would this concept work with your current process? What do you like about it, and what do you wish were different?

Conclusion

- From all the solutions that you’ve seen, what do you like, and what do you wish were different?
- Is there anyone else you know who you think it might be helpful for us to talk to?
- Any questions for us?


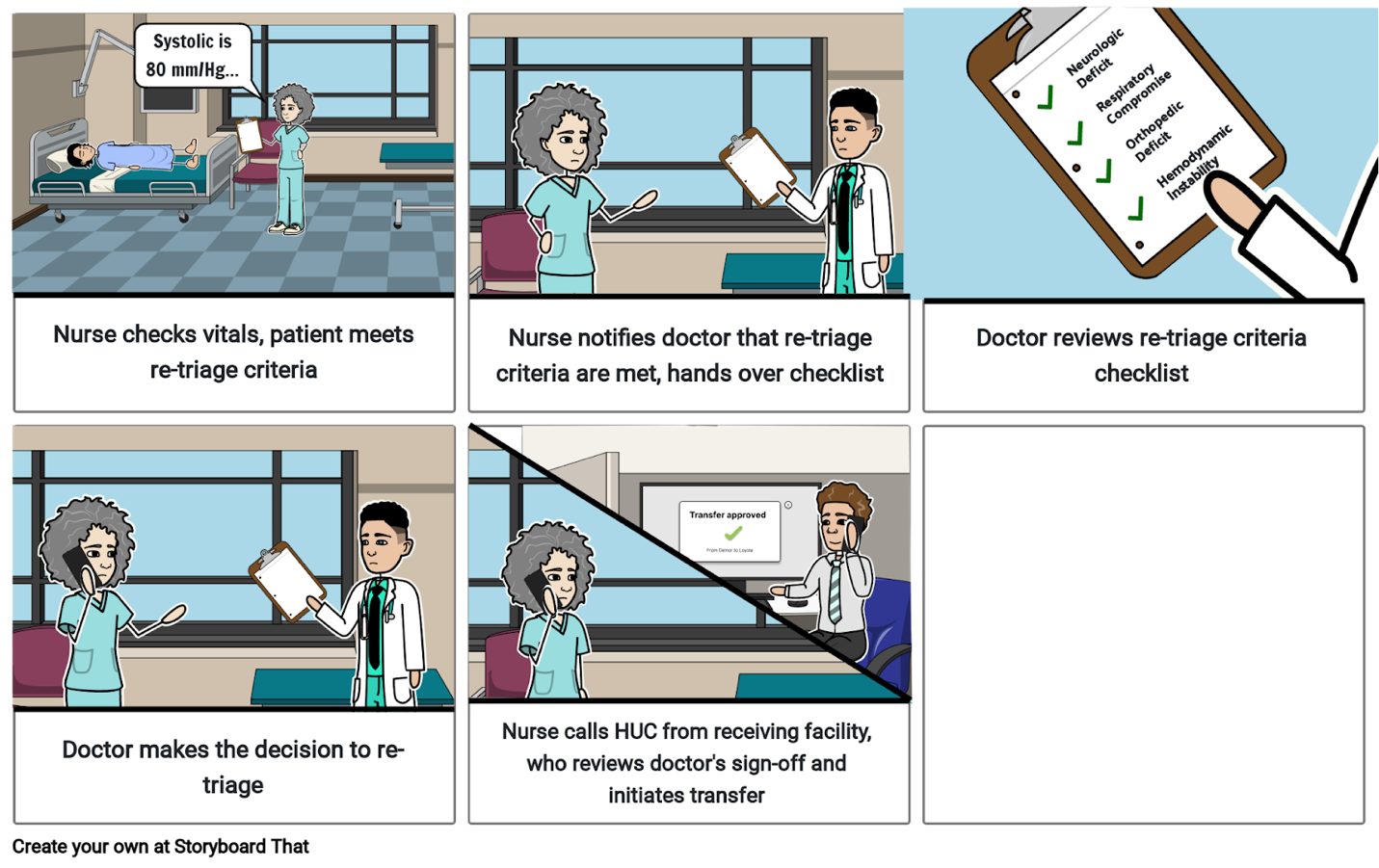


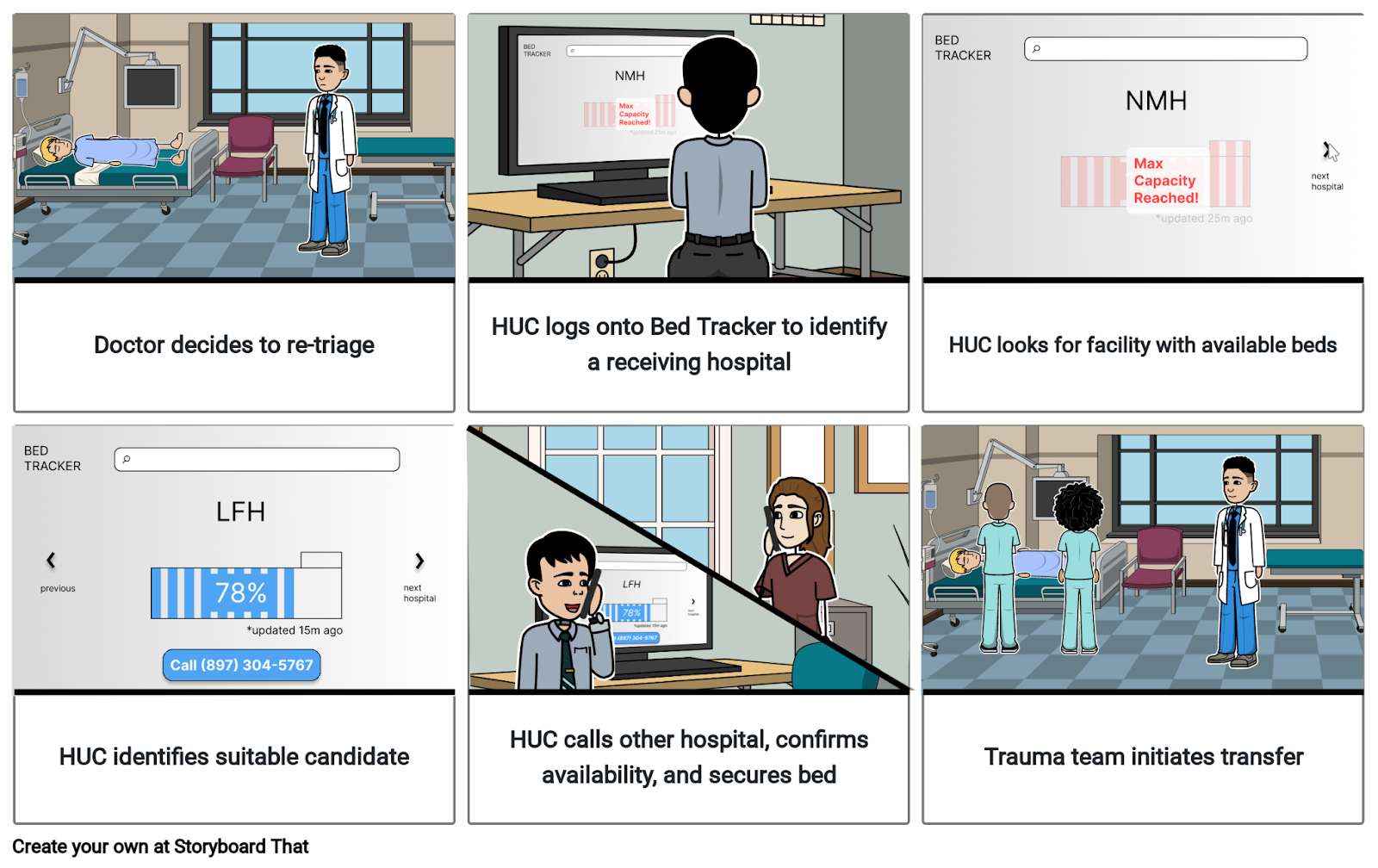


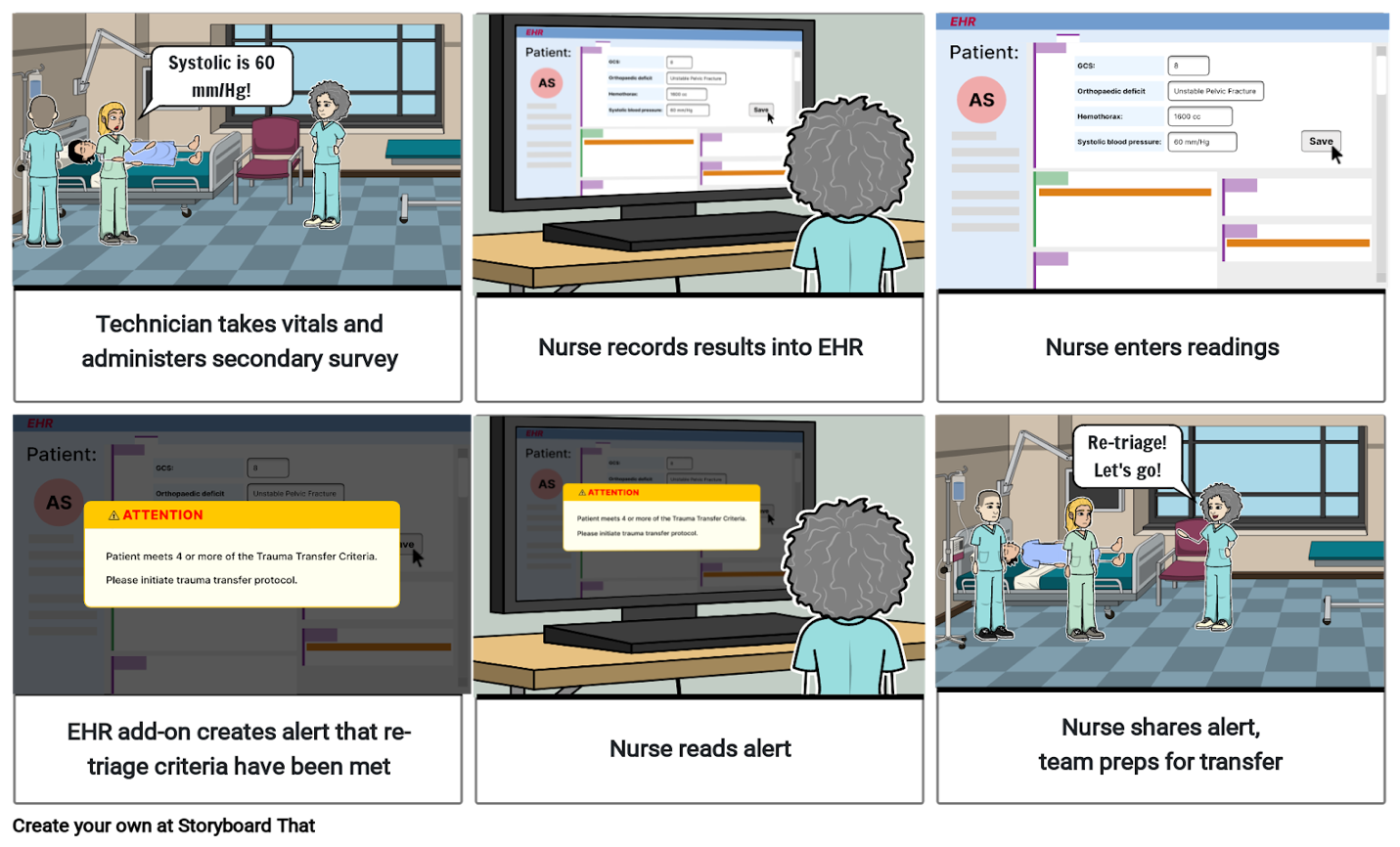

Supplement: Multimedia Appendix 3 [file jmir-v27-e70846-s003.docx]
