# Supplementary material for: User-Centered Design of Trauma Systems Solutions for Retriage of Patients With Injury: Mixed Methods Study
Source: J Med Internet Res. 2025 Aug 27;27:e70846. doi: 10.2196/70846 (PMC12381891; doi:10.2196/70846)
Supplement: Multimedia Appendix 4 [file jmir-v27-e70846-s004.docx]

| **Phase** | **Codes** | **Definition** | | | **Example** |
| --- | --- | --- | --- | --- | --- |
| **Phase I** | **ICU vs. ED Bed Capacity** | | Comparison between bed capacity in ED vs ICU in the context of re-triage for more granularity about bed information displayed in bed tracker | “Some questions I would have as when you say capacity, what do you mean? Do you mean ER capacity, do you mean OR capacity? Do you mean ICU bed capacity? Do you mean floor bed capacity?” – Trauma Coordinator, High-Level Receiving Center | |
| **Phase I** | **Overview of Trauma Center Layout** | | Description of interviewee’s trauma center | “…at [receiving trauma center], we're unique because my ICU and Resuscitation Bay are 20 feet apart. So I know what my ICU capacity is and I know what my ER capacity is very quickly. These other trauma centers, or everyone else that you named, their trauma bays are built within their ER.” – Trauma Coordinator, High-Level Receiving Center | |
| **Phase I** | **Interaction with Separate Hospital** | | Highlighting disparities in the processes of completing re-triage processes between different trauma centers. | “Where we've had pediatric patients injured, 14 and 15 years old, that we wanted to get to a pediatric facility and the pediatric facility is like, ‘They're really not peds.’ But for our purposes, they are. They couldn't be admitted in our hospital because it's not peds... Because they are peds. But from the pediatric standpoint, it's not peds, which is another kind of conversation. But just that's something we've also run into with that transferring process. And it really is those weird middle ages where they definitely go... 16 to 18 that they'll say, ‘Not really peds.’ But we've had them as young as 14 where they're like, ‘They're not really a pediatric patient.’” – Trauma Nurse, Low-Level Sending Center | |
| **Phase II** | **Rapidly Changing Bed** **Capacity** | | Analyzing the disconnection between what the transfer center personnel perceived to be the case versus what's actually going down in the ED is because of a lack of real-time update. | “I think what you're alluding to with bed available on a hospital-wide basis, you're right…I can tell you exactly how many patients I have. So, that's more realistic, but I don't know if that's applicable across other institutions. So, that's where, like, if you call me, I could update that every four to six hours. It might be adding a burden somewhere, but every four to six hours I can tell you, ‘Yeah, I've got three ICU beds and I've got 10 beds for [trauma] transfers.’” – Trauma Surgeon, High-Level Receiving Center | |
| **Phase II** | **Difference between Capacity vs. Ability to Accept Transfers** | | There's a need to facilitate effective communication between medical facilities and ensure efficient patient transfers. It is crucial to maintain accurate and up-to-date information to avoid unnecessary delays and optimize patient care. | “I need to know are they accepting transfers, yes or no. Put it on the onus of the trauma center to say, am I accepting transfers, yes or no? Because I'm assuming that me as the accepting center, I'm either going to have to have my charge nurse, my attending, I'm going to have to be filling this out and update this either every four hours, eight hours as needed.” – Trauma Coordinator, High-Level Accepting Center | |
| **Phase II** | **Transfer Acceptance Requirements** | | Requirements that determine whether a given facility is able to accept a re-triage candidate. | “Consider re-triage. If it's vital signs, all you have is vital signs and hemothorax, unstable pelvic fracture, GCS of eight. So we may keep a GCS of eight. Orthopedic, we usually will keep those patients unless it's something our ortho cannot handle. Hemothorax would definitely be transferred out. Systolic blood pressure, we would have to stabilize. And if we got them stabilized, we could very possibly keep them.” – Trauma Coordinator, Low-Level Sending Center | |
| **Phase II** | **Who knows which beds are available at one time** | | Descriptions of individuals with knowledge of bed capacity in their service that would need to be aggregated for a bed tracker | “At [low-level sending hospital], as far as bed availability…we have a general idea of what we have available as far as when they're being transferred out to another facility. Generally, the providers, based on what we're seeing, whether it ends up being the surgeon or one of our ED providers, when we're trying to transfer them out, they usually look to region nine first.” – Trauma Coordinator, Low-Level Sending Hospital | |
| **Phase II** | **Authority to initiate acceptance of re-triage candidate** | | People who have the authority to make the call to decide if they are accepting transfers or not. | “I'll put it this way, when I feel a bed crunch in my own trauma recess area, I am often... I don't want to say overruling the nursing supervisor, but I'm trying to barter, make exchanges, and try and explain to them, 'No, no, here is logistically...’ I try and tell them what they could and should do to accommodate when we do have a mass casualty at hand, be like, ‘Hey, why don't we put this patient in this ICU, this patient in this ICU.’ – Trauma Surgeon, High-Level Receiving Center | |
| **Phase II** | **Workaround strategy for bypass laws** | | Highlighting how to make the users/doctors adapt to the re-triage workflow. | “And so you mentioned that the person who might be best positioned to actually input this data from a receiving hospital's perspective would be the attending?” – Trauma Coordinator, High-Level Receiving Center | |
| **Phase V** | **Applicability to Front-End User Interaction with Re-Triage Checklist** | | How re-triage intervention would apply to an interviewee’s current workflow/how they would interact with the intervention/app/etc. | “If the patient is that severe and meets the checklist and gets transferred right as I'm walking in the door, then it's sort of a waste of my time to drive in to see the patient because then the patient's automatically just leaving…who's doing the checklist and how is that going to change the role of the trauma surgeon in the community setting?...That's what the state mandates…When it's a Level 1, I have to drive in and examine the patient. That's the whole point of trauma evaluation by a trauma surgeon in the trauma center. So I think a checklist is great, but then it's not supposed to be the end all be all.” – Trauma Surgeon, Low-Level Trauma Center | |
| **Phase V** | **Applicability to Front-End User Interaction with Bed Tracker** | | How re-triage intervention would apply to an interviewee’s current workflow/how they would interact with the intervention/app/etc. | “… if you're in your own system, then it's different and people are familiar with resources and if you think, okay, the patient needs a splenic embolization or an orthopedic procedure, you don't have the right people where you are, getting them to the correct place quickly is important. And I suppose that might help get them to the right place, but it would be tricky across systems.” – General Surgeon, Sending Non-Trauma Center | |
| **Phase V** | **EMResource Use in Current Workflow** | | Overview of how EMResource is or could be used in the current-state workflow | “I asked them to make a report internally in Epic that mirrors the fields that we need to complete in EMResource to report that bed availability. So what I do is I can run the report and get a live feed of all of those bed counts, so it's not a manual process. And then we have dual, you're only seeing me on one screen, but I have two large monitors here. So we basically put the report up on the one screen and we have EMResources on the other and we just copied the numbers right over. So we have fields that match each one.” – Bed Manager, Non-Trauma Center | |
| **Phase V** | **EMResource Use with Bypass Laws** | | Description of how bypass laws in Illinois can impact transfer decisions and how EMResource is used when trauma centers are at capacity. | “…looking at this, and this is coming from a program manager point, because it's with the bypass laws of Illinois, everyone worries about the bypass laws.” – Trauma Coordinator, High-Level Receiving Center | |
| **Phase V** | **Determining correct types of information to provide on bed capacity** | | Highlighting the importance of integrating relevant information for effective transfer decision making, considering the potential impact of excessive information on decision quality. | “I think if you got too granular with the information and you start really getting units specific, we have a saying here, backseat quarterback, bed manager, do you want to be a doctor or do you want to be a bed manager?…So I think that's where I just mean if you really listed every unit…and how many just surgical beds and how many, every type of ICU. We have four ICUs, not including peds and NICU here at [sending, non-trauma center]…That might just be too much information that what I wouldn't want to see happen to any transfer center staff anywhere in the United States is someone calls them and says, well this site says that you have capacity so you must have capacity and therefore you must take my patient.” – Bed Manager, Non-Trauma Sending Center | |
| **Phase V** | **Feedback on indicating Ability to Accept Transfers in Bed Tracker** | | Suggestions/insight re: how a potential intervention could/should function to maximize feasibility | “I do like adding filters to it if possible. I like the electronic checklist, but the only thing that we need to add to that is that it should be more of a notification rather than an automatic decision maker that there has to be a safety, and there has to be a human confirmation, at the end of that checklist. The checklist is going to say, yeah, this patient might be appropriate for a transfer, not that the patient is appropriate for a transfer. And then the physician has to confirm that yes, this patient's appropriate for transfer and then we initiate the transfer. Cause if you're going to automate it, you're going to be transferring patients that don't need to be transferred.” – General Surgeon, Low-Level Sending Center | |
| **Phase V** | **Ownership of Bed Tracker Updating** | | Descriptions of who is responsible for updating a hospital’s bed capacity in a tracker/EMResource | “…your charge nurses in the ER, their main focus is going to be EMResource because that is the legal, that the holy grail. So if they have to go on bypass or if they have to do anything, their job is keeping EMResource up to date. So that is going to be their main focus.” – General Surgeon, Low-Level Sending Center | |
